# Supplementary material for: Parents’ or Guardians’ Decisions on Human Papillomavirus Vaccine Acceptance for School Children in a Southern Province of Thailand: A Mixed-Method Study
Source: Vaccines (Basel). 2025 Dec 31;14(1):53. doi: 10.3390/vaccines14010053 (PMC12846666; doi:10.3390/vaccines14010053)
Supplement: Supplementary file 1 [file vaccines-14-00053-s001.zip › vaccines-4025039-supplementary.pdf]

Supplementary file

Section S1. Quantitative study

Supplementary Table S1 Responses of participants to individual knowledge questions regarding HPV and its vaccine.

Questions	correct n (%)	incorrect n (%)	Median (IQR) of Acceptance of HPV Vaccine for their children			P value
			Total n=943	No n=280	Yes n=663	
1. HPV stands for Human papillomavirus.	854 (90.5)	90 (9.5)	1 (1,1)	1 (1,1)	1 (1,1)	0.008*
2. HPV cannot cause cancer in males.	552 (58.5)	392(41.5)	1 (0,1)	1 (0,1)	1 (0,1)	0.846
3. HPV can cause cancers of the oral cavity/pharynx, anus, vagina, and penis.	798 (83.6)	146(16.4)	1 (1,1)	1 (1,1)	1 (1,1)	0.043*
4. Most HPV infections are transmitted through sexual contact, and condoms do not offer complete protection.	599 (63.5)	345(36.5)	1 (0,1)	1 (0,1)	1 (0,1)	0.467
5. After contracting a cancer-causing strain of HPV, the body is unable to eliminate the virus.	629 (66.6)	315(33.4)	1 (0,1)	1 (0,1)	1 (0,1)	0.084
6. After contracting HPV, it can take more than 10 years for cancer to develop.	600 (63.5)	344(36.5)	1 (0,1)	1 (0,1)	1 (0,1)	<0.001*
7. The HPV vaccine and the cervical cancer vaccine are different types of vaccines.	403 (42.7)	54 (57.3)	0 (0,1)	0 (0,1)	0 (0,1)	0.234
8. The HPV vaccine can only prevent cervical cancer.	445 (47.1)	499(52.9)	0 (0,1)	1 (0,1)	0 (0,1)	0.033*
9. There are several types of HPV vaccines currently available, each protecting against different strains of HPV.	734 (77.8)	210(22.2)	1 (1,1)	1 (1,1)	1 (1,1)	0.277
10. The HPV vaccine is safe and effective in preventing cancers caused by HPV infection.	763 (80.8)	181(19.2)	1 (1,1)	1 (1,1)	1 (1,1)	0.003*
11. Children aged 9–14 years require only 2 doses of the vaccine; those aged 15 and older require 3 doses.	720 (76.3)	224(23.7)	1 (1,1)	1 (0,1)	1 (1,1)	<0.001*
12. The HPV vaccine can be administered to both males and females.	719 (76.3)	225(23.7)	1 (1,1)	1 (0,1)	1 (1,1)	0.226
13. The HPV vaccine is most effective when given before the first sexual intercourse.	705 (74.7)	239(25.3)	1 (1,1)	1 (0,1)	1 (1,1)	<0.001*
14. If someone has already had sexual intercourse, they can no longer receive the HPV vaccine.	567 (60)	377 (40)	1 (0,1)	1 (0,1)	1 (0,1)	0.247

Note: a. Scoring method: 1=correct answer, 0=incorrect answer.

b. Cut-points: 11.21-14=High, 8.41 -11.20=Moderate, ≤8.40=low level of knowledge.

*p<0.05, Wilcoxon rank-sum test

Supplementary Table S2. Association of access to HPV vaccination information or services with parental acceptance of HPV Vaccine for their children

Questions	Yes n (%)	No n (%)	Median (IQR) of Acceptance of HPV Vaccine for their children			P value
			Total n=943	No n=280	Yes n=663	
1. Have you ever heard of or been aware of HPV vaccine?	575 60.9)	369 (39.1)	1 (0,1)	0 (0,1)	1 (0,1)	<0.001*
2. Have you ever received a recommendation from a healthcare professional for your child to receive a HPV vaccine?	416 (44)	528(56)	0 (0,1)	0 (0,1)	0 (0,1)	<0.001*
3. Are you aware of the government-supported HPV vaccination program?	428(45.3)	516(54.7)	0 (0,1)	0 (0,1)	1 (0,1)	<0.001*
4. Do you know where the HPV vaccine is available in your area of residence?	366(38.8)	578(61.2)	0 (0,1)	0 (0,1)	0 (0,1)	<0.001*
5. Do you face any barriers to have your child to receive a HPV vaccine? (e.g., lack of information, cost of vaccination, or distance to the vaccination site)	436(46.2)	508(53.8)	1 (0,1)	0.5 (0,1)	1 (0,1)	0.107

Note: Question 1-4, Yes=1, No=0; Question 5, Yes=0, No=1

*p<0.05, Wilcoxon rank-sum test

Supplementary Table S3. Parental attitudes and concerns about HPV vaccination

Questions	Answer					Total
	Strongly agree n %)	Agree n (%)	Neutral n (%)	Disagree n (%)	Strongly disagree n (%)	
1.Do you think HPV vaccine is important for preventing cervical cancer, vaginal cancer, penile cancer, oral and oropharyngeal cancer, and anal cancer?	438(46.5)	354(37.7)	126(13.4)	18(1.9)	5(0.5)	941 (100)
2. Do you think the bivalent and quadrivalent HPV vaccines are sufficient for preventing HPV-related cancers?	196(20.9)	477(50.7)	191(20.3)	59(6.3)	17(1.8)	940 (100)
3. Do you think males should be encouraged to receive a course of HPV vaccine?	295(31.4)	384(40.8)	205(21.8)	41(4.4)	15(1.6)	940 (100)
4. Do you think the government should provide free HPV vaccination for both girls and boys before they are at risk of sexual activity as a health promotion measure?	431(45.9)	367(39)	113(12)	22(2.3)	8(0.8)	941 (100)
5. Do you think the current campaigns or public communications regarding HPV vaccine are insufficient?	362(38.5)	392(41.7)	162(17.2)	18(1.9)	7(0.7)	941 (100)
6. Are you concerned about potential side effects after receiving the HPV vaccine?	318(33.8)	389(41.3)	204(21.7)	20(2.1)	10(1.1)	941 (100)

Questions	Answer					Total
	Strongly agree n %)	Agree n (%)	Neutral n (%)	Disagree n (%)	Strongly disagree n (%)	
7. Are you concerned about the cost if you would like your child to receive HPV vaccine from a private provider (approximately 6,000–18,000 baht, typically paid in 2–3 installments per person)?	25(4.8)	263 50.7)	154(29.7)	54(10.4)	22(4.2)	518 (100)

Note: Questions 1–4, which assessed perceptions of the vaccine's benefits (score 5=strongly agree to 1=strongly disagree). Questions 5–7, which assessed concerns about side effects and cost (score 1=strongly agree to 5=strongly disagree).

Supplementary Table S4. Association of parental attitudes and concerns about HPV vaccination with parental acceptance of HPV vaccine for their children (*p<0.05, Wilcoxon rank-sum test)

Questions	Median (IQR) of Acceptance of HPV Vaccine for their children			P value
	Total n=943	No n=280	Yes n=663	
1. Do you think the HPV vaccine is important for preventing cervical cancer, vaginal cancer, penile cancer, oral and oropharyngeal cancer, and anal cancer?	4 (4,5)	4 (3,5)	5 (4,5)	<0.001*
2. Do you think the bivalent and quadrivalent HPV vaccines are sufficient for preventing HPV-related cancers?	4 (3,4)	4 (4,4)	4 (3,4)	<0.001*
3. Do you think males should be encouraged to receive the HPV vaccine?	4 (3,4)	4 (4,5)	4 (3,5)	<0.001*
4. Do you think the government should provide free HPV vaccination for both girls and boys before they are at risk of sexual activity as a health promotion measure?	4 (3,5)	5 (4,5)	4 (4,5)	<0.001*
5. Do you think the current campaigns or public communication regarding the HPV vaccine are insufficient?	4 (3,5)	4 (4,5)	4 (4,5)	<0.001*
6. Are you concerned about potential side effects after receiving the HPV vaccine?	4 (3,5)	4 (4,5)	4 (4,5)	0.037*
7. Are you concerned about the cost if you want your child to receive the HPV vaccine from a private provider? (approximately 6,000–18,000 baht, typically paid in 2–3 installments per person)	4 (3,5)	5 (4,5)	4 (4,5)	<0.001*
**Average of total score**	3(2.9,3.3)	3.1 (3,3.3)	3.1 (3,3.3)	< 0.001*

Note: Average score: 4-5 = Positive, 3=Neutral, <3 =Negative attitudes

*p<0.05, Wilcoxon rank-sum test

Supplementary Table S5. Baseline characteristic of the interviewed participants

No.	Sex	Relationship to the child	Religion	Education	Type of children's school	Presence of healthcare professional	Acceptance of HPV Vaccine for their children	Acceptance of HPV vaccine for their children#
1	female	parents	Buddhism	>Bachelor	PS	Yes	Yes	Male: (-)
2	female	parents	Buddhism	Bachelor	CGS	No	No	Male: (-)
3	female	parents	Buddhism	<Bachelor	LCS	No	Yes	Female: (+)<br>Male: (-)
4	female	parents	Buddhism	>Bachelor	PS	No	Yes	Female: (-)
5	female	parents	Buddhism	Bachelor	CGS	N0	Yes	Female: (+)
6	female	parents	Buddhism	<Bachelor	LGS	No	Yes	Female:(+)<br>Male:(-)
7	female	parents	Buddhism	Bachelor	LGS	No	Yes	Female:(+)
8	female	parents	Islam	<Bachelor	CGS	No	Yes	Female:(+)
9	female	Guardians (Aunt)	Buddhism	<Bachelor	CGS	No	Yes	Male: (-)
10	female	parents	Buddhism	>Bachelor	PS	Yes	Yes	Male:(+)<br>Female:(+)
11	male	parents	Buddhism	>Bachelor	CGS	No	Yes	Female:(+)

Abbreviations: CGS, central governmental school, LGS, local governmental school, PS, private school

(+): received vaccine, (-) did not receive vaccine

Section S2. Qualitative study

The detailed content of quotations from the in-depth interviews

A. Knowledge, Information and attitudes

The participants still misunderstood regarding HPV vaccine, particularly in the following areas:

1. Only female was the target population needing of HPV vaccination

There is an understanding that this vaccine is solely for women. Some believed that it could be given to men too, but the primary efficacy was on women. Awareness of the vaccine's role in preventing HPV-related diseases in men is almost non-existent.

"At first, I understood that it was administered only to women, but later I found out that it could also be given to men to prevent HPV infection and cancers related for their sexual partners." (P1)

"It's for prevention of cervical cancer if it was given in pre-teens. I didn't know it clearly because I didn't have a daughter. So, I wasn't that interested." (P2)

"Initially, I didn't think it could be administered to men, but later I knew from medical pamphlets and websites that it also could be given to men to prevent their sexual partners. They

said it was a sexually-transmitted disease, and HPV could be transferred to women leading to cervical cancer many years later.” (P1)

“I vaguely know that it will be given to the sixth-graders to prevent cervical cancer in girls, but I don’t know that it is for boys too.” (P2)

“It’s for women, but how about the kids aged 10-13 years? I’m not sure whether they are too young for the vaccine. If it is related to the disorders of uterus but men have no uterus, should men be vaccinated?” (P3)

“For men, I think it may not have any clear effects on them.” (P1)

“I never paid much attention on the vaccine because I don’t have a daughter. I didn’t know that boys could receive the vaccine too.” (P2)

“By the common name of the HPV vaccine (cervical cancer prevention vaccine), it sounds like it should be prescribed to only females. Since males have no uterus, then, for what kind of cancer it will prevent?” (P3)

“Cervical cancer vaccine’ makes me don’t think that men can get it. Men don’t have uteruses, right? (P4)

“Very few boys are vaccinated. Their parents will question why I should take my son for it.” (P10)

“My son hasn’t been vaccinated because I don’t know boys can receive it. At school, only girls receive the vaccine. If boys benefits from receiving it, then, it should be given to them as well.” (P11)

2. Information about safety or side effects of HPV vaccine was inadequately available

There was still lack of clarity regarding the side effects of vaccines. Parents had not yet decided to have their male children to receive the vaccine due to the concerns about possible side effects. The concerns resembled the case of COVID-19 vaccine during the past pandemic.

“I’m also concerned about the adverse effects like those we have heard about COVID-19 vaccine. I, myself, think HPV vaccine is a newly-developed vaccine. I am worried about its side effects. Payment for vaccine cost is not an issue, but I want to know what side effects the children may experience after being vaccinated.” (P2)

“The main issue is the lack of correct understanding. Some people only know that it is a vaccine, but if I allow my children to be vaccinated, what the adverse events will happen to them?” It’s because of the lack of knowledge, most people tend to think negatively a lot. They don’t know how the vaccination really affects the body’s functions. They gather a lot of opinions from many people and believe in several harms unreasonably resulting in so much concerns. If no one comes to provide clear-cut and correct information, they possibly believe in what they have been known. They usually say, ‘Well, this is what I’ve known’ and they are hesitant to have their children vaccinated.” (P7)

“Just like COVID-19 vaccine, we saw some people became paralyzed, and most people thought it was caused directly by the vaccine.” (P9)

3. Unclear understanding and lack of public information about the benefits of the vaccine

“Most of what I knew about the vaccine were from websites or the leaflets distributed by private hospitals. I learnt a little about the vaccine myself, I didn’t study it in depth.” (P1)

“Hospitals provide information about the vaccine, but I don’t pay any attention on them. Only until the healthcare providers would give the vaccine to my child—then, I started to take attention.” (P3)

“At the university hospital, there were many pamphlets informing the different health topics, including HPV and its vaccine. I didn’t read the details, but I knew that the nurses vaccinated those who adopted it.” (P4)

“I remembered that I had heard about it in short clips through the online social medias. I didn’t pay much attention at that time but I was surprised to learn there was a vaccine for prevention cervical cancer. Since I am a man, I am not interested in the vaccine but I’ve been known about it more over the past year.” (P11)

"Schools are facilitating the students to receive vaccine—both in primary and secondary levels. My daughter was in secondary-level school, and they asked her to receive the vaccine. I searched the information from online websites or Google platform because I wanted to know more about the vaccine such as what was it for and what it could prevent." (P8)

"It will be better to have a doctor, or the best a pediatrician, to discuss about vaccine benefits and safety to the parents. Parents will rely mostly on a doctor than the currently available online information sorces." (P2)

4. The timing of effective HPV vaccination.

"I think proper time for receiving the vaccine should be after menstruation starts. For boys, maybe from the age 15 on. I think their sex hormones are starting to fully develop. I strongly agree that it's better to receive the vaccine once menstruation starts and before the children become sexually active." (P2)

"I think it should be during puberty. For girls, probably when they start menstruation." (P3)

"Whether children in secondary school are appropriate for vaccination? In my opinion, secondary school children seem more appropriate than those in primary school which are too young. I think it's better to wait until their sex hormones are fully developed before getting the vaccine." (P6)

"I think it should be given only to the girls who have already started menstruation. Once menstruation begins, the child is capable of pregnancy, so their reproductive system has fully developed." (P7)

"I think it should be before menstruation, because after it starts, the uterus changes, right? That's just my thought—I don't have any scientific knowledge about this issue. But, I think HPV vaccine may help protect the uterus, ovaries, and fallopian tubes. That's why I think it should be given." (P8)

5.The experience with HPV vaccination of the others

"I felt a bit worried initially, because no one around us had ever received the vaccine yet. I didn't know if there would be an allergic reaction. It was like a new vaccine, so I feared of possible side effects. I would be less concerned about the vaccine, if there are rarely side effects occured." (P8)

"The younger sibling was vaccinated first before the older one at their school. Since no side effect occurred in the younger one, we let the older one had it too. Only a little of a headache experienced afterward, but it resolved finally. I'm not so worried, but it seems to affect people differently. So, now both of our children have completed two doses of HPV vaccines." (P9)

"I have already received the quadrivalent vaccine when it was launched around 2012 or 2013. I believe in the efficacy of the vaccine in protection against cancers." (P10)

"I will not allow my children to be vaccinated if I know any adverse effects occurred in the people who have been vaccinated." (P3)

B. Barriers of Access to Cervical Cancer Vaccination

The participants reported several barriers in access to the HPV vaccine, which can be categorized into the following key areas:

1. Limited access to a reliable health information source

"There are village health volunteers and also community caregivers in our villages. It would be good if the local health agencies could come to the villages and communicate through village leaders or loudspeakers. Todays, people do not accept the vaccine unless they have experienced the illness. I

recommend to suggest vaccination program for boys in the campaign as well. Cooperative working with the local leaders to disseminate information is essential. Sunday or late morning will be better time to meet the villagers with the introduction of vaccine knowledge from the community leader and healthcare agencies. (P3)

"I wish the local public health officers will declare the definite governmental support for children to receive this vaccine." (P4)

"They should promote HPV vaccine to both parents and children, especially the girls who are studying in grades 5 or 6. Sharing the information in the community through a meeting on public holidays, will be helpful. There's a healthcare center nearby, but the healthcare staff haven't come to visit our area." (P6)

"We didn't know the vaccine was beneficial to prevent cervical cancer. We also didn't understand the details—like how many strains it could protects against. I'd like the healthcare providers to organize knowledge-sharing sessions so we can gain a clearer understanding." (P7)

"Hospitals should run campaigns and educate the parents—then more children will be vaccinated. People in the community don't really know much does the vaccine cost per course. If they were informed about the cost, more people may accept it. Community-based sessions during late mornings on the weekends will be ideal. Home visits may be more helpful than large group events in that an individual can freely ask questions for clear understanding. Social media is also an useful route for information distribution, but it needs official verification or the information should be distributed by the official sectors." (P8)

"I often see medical students come to visit our village like village health volunteers, but they never discuss this topic. I think if they can promote this vaccine by explaining its benefits and adverse effects, it's a more direct form of outreach to the target people." (P11)

Based on the background and experiences of health promotion, the community healthcare providers available in a local area are able to handle the vaccine promotion, if they are additionally trained on HPV vaccine knowledge." (P1)

2. The campaigns on HPV vaccination is less strong

"A vaccination program should be should be suggested to both parents and their kids—especially girls who are studying in grades 5 or 6. It would be best to do the direct approach individually by the healthcare provider. (P6)

"Campaigns can be done in school-organized meeting events in which the parents are involved. And through the current social medias available such as TikTok, Facebook, or Instagram are the way to reach the target teenagers directly." (P7)

"Community or house-to-house visits on the free time of the community people will be best suited action for vaccine campaign. Home visits help exert more impact than big group sessions." (P8)

."A doctor or healthcare provider is the most reliable person to discuss side effects vs. benefits of vaccine to facilitate parents' decision to accept vaccine for their young children." (P3)

3. High Cost and limited governmental funding support.

"It should not more than 500–1,000 THB per dose of vaccine which can be affordable by a majority of people. Also, I need to know more about the potential side effects of the vaccine." (P2)

"Not over than 1,000 THB per dose." (P3)

"Around 2,000 THB per dose that I can afford." (P4)

"The vaccine cost should be comparable to that of influenza vaccine, which was used to be 700–900 THB, but now it's around 450–500." (P5)

"I can afford below 2,000 THB for the full course of vaccine." (P6)

"I think vaccine cost should not be more than 1,000 THB." (P7)

"If possible, the cost should under 900 or 1000 THB." (P8)

"I know the vaccine has high cost. I don't know the exact price, but I heard it's very costly." (P9)

" I has been told recently that vaccine cost can be paid by applying to national healthcare coverage scheme. This is very favorable to help most people who earn less income." (P5)

Remark: (THB, Thai Baht. 1 USD = 31.07 THB on 27 Dec 2025)

4. Geographical barriers in accessing to HPV vaccine

"It's difficult to receive the vaccine because a hospital or healthcare center is far away from home. The district hospital is located over ten kilometers away, while the provincial hospital in Hat Yai or Songkhla is over one hundred kilometers away from my home. If the vaccine isn't brought to the villages by hospital staff, it's very difficult to access. Some people are inconvenient to travel such a long distance to receive the vaccine." (P4)

"I think school-base administration of HPV vaccine to the school children is more feasible than having people to receive the vaccine in a hospital." (P6)

Section S3. Good Reporting of A Mixed Methods Study (GRAMMS)

**Guideline**	**Section: page**
Describe the justification for using a mixed methods approach to the research question	Introduction; p 2<br>Study design and setting: p 2<br>Strengths and limitations: p 12
Describe the design in terms of the purpose, priority and sequence of methods	Study design: p 2<br>Data collection: p 3-4
Describe each method in terms of sampling, data collection and analysis	Sampling techniques: p 3<br>Data collection: p 3-4<br>Data analysis: p 4
Describe where integration has occurred, how it has occurred and who has participated in it	Study design and setting: p 2<br>Data collection: p 3-4
Describe any limitation of one method associated with the present of the other method	Strengths and limitations: p 12-13
Describe any insights gained from mixing or integrating methods	Discussion: p 12

O'Cathain A, Murphy E, Nicholl J. The quality of mixed methods studies in health services research. J Health Serv Res Policy. 2008;13(2):92-98.
